# Optimal Strategies to Reduce Inappropriate Implantable Cardioverter-defibrillator Shocks

**DOI:** 10.19102/icrm.2019.100403

**Published:** 2019-04-15

**Authors:** Blake E. Fleeman, Ryan G. Aleong

**Affiliations:** ^1^University of Colorado, Aurora, CO, USA

**Keywords:** ICD, implantable cardioverter-defibrillator, inappropriate therapy, sudden cardiac death

## Abstract

Since the widespread implementation of implantable cardioverter-defibrillators (ICDs), their effectiveness in various situations has become well-established. However, despite many advances in both the technology and its utilization, inappropriate therapy remains a risk. Here, we review ICD shocks, their effect on outcomes, and current methods to reduce inappropriate therapy, finding overall that inappropriate ICD shocks are common and associated with adverse outcomes. However, strategies do exist to minimize inappropriate shock rates including device selection and programming, medication, catheter ablation, and remote monitoring. Overall, ICDs are useful in reducing the risk of sudden cardiac death, but many patients with an ICD will receive an inappropriate shock. Understanding strategies to prevent inappropriate shocks is crucial to improving the care of patients with ICDs.

## Introduction

Implantable cardioverter-defibrillators (ICDs) have transformed the management of sudden cardiac death. Initially, ICDs were utilized only in a select few kinds of patients with risk factors and inducible or prior ventricular tachycardia (VT) or ventricular fibrillation (VF). However, in 2004, the Multicenter Automatic Defibrillator Implantation Trial (MADIT II) investigators demonstrated a mortality benefit at one year for certain patients receiving prophylactic ICD implantation.^1^ Since the completion of this and other large randomized trials, the use of ICDs has increased and led to their widespread implementation. Overall, their effectiveness in various situations has become well-established. However, despite many advances in both the technology and methods of incorporation into treatment, inappropriate therapy remains a risk.

## The harm of inappropriate shocks

Although ICD therapy is potentially life-saving, device shocks are not benign and the cost of inappropriate shocks in particular may be high. Experiencing an ICD shock is extremely uncomfortable and can be a traumatic experience. Research has indicated ICD shocks to be associated with a reduced quality of life.^2^ Furthermore, receiving ICD shocks may be a risk for other morbidities and mortality. For example, Poole et al. was purportedly the first to report that both appropriate and inappropriate shocks were associated with a higher mortality rate in heart failure patients.^3^ Separately, in MADIT II and the Sudden Cardiac Death in Heart Failure Trial (SCD-HeFT), there was an association between receiving shocks and a subsequent two- to fivefold increased risk of death.^1,4^ In another study, receiving even a single inappropriate shock was associated with an increased risk of mortality, with a hazard ratio (HR) of 1.6.^5,6^ In the same cohort, mortality risk increased progressively with subsequent shocks, reaching an HR of 3.7 after having received five inappropriate shocks.

There is also a high cost in the form of health care utilization and spending attributable to inappropriate shock. In a recent analysis of a cohort of 10,266 patients, health care expenditures following episodes of inappropriate shock were high—similar to the costs present following appropriate shocks. Cardiovascular procedures were frequently performed even after inappropriate shocks, including echocardiograms in most and coronary angiography in 51% of patients, respectively.^7^

Interestingly, the cause of an inappropriate shock may affect the risk it poses to the patient. It is unclear as to whether the increased risk of harm following an inappropriate shock is due to the shock itself or to the underlying cause. In one prospective trial, 1,411 patients with ICDs were followed for a median of three years. Notably, the risk of mortality was highest in patients with appropriate shocks. However, among those with inappropriate shocks, the risk of mortality was significantly higher for those with shocks due to atrial fibrillation (AF) as compared with in those who experienced shocks due to lead failure. There was a worse prognosis for those suffering multiple shocks due to AF (HR: 1.4), but there was no effect on mortality for those with one or more shocks occurring as a result of lead failure.^8^ These findings may support the notion that the shock itself is not the cause of harm but instead is a marker of the underlying pathology.

## Frequency of inappropriate shock

Inappropriate shocks arising from ICDs are unfortunately not rare. The rate of inappropriate shock was reported to be as high as 13% to 17% in the era of SCD-HeFT and MADIT-II.^1,4^ However, as the risk and frequency of inappropriate shock have been appreciated more in recent years, increasing attempts have been made to limit this phenomenon, and these efforts may in fact be having an effect. A recent meta-analysis that included 16 trials published between the years 2002 and 2015 and which reported on 14,696 patient-years of cumulative follow-up identified an annual inappropriate shock rate of 6.4% that progressively lessened over time.^9^ With the application of focused strategies, this rate may potentially be even further improved. Inappropriate shock probabilities of 5% and 3%, respectively, were seen in the Multicenter Automatic Defibrillator Implantation Trial—Reduce Inappropriate Therapy (MADIT-RIT) and Avoid Delivering Therapies for Nonsustained Arrhythmias in ICD Patients III (ADVANCE III) trials.^10,11^ Separately, in the PainFree SmartShock Technology (SST) study, in which patients received a device with a combination of detection algorithms as well as programming strategies to minimize inappropriate shocks, an annualized rate of inappropriate shocks of only 1.9% was observed.^12^ This trend highlights the importance of understanding the causes of inappropriate shocks in order to more effectively pursue strategies in order to minimize the risk of such.

## Causes of inappropriate shock

**Figure 1:**
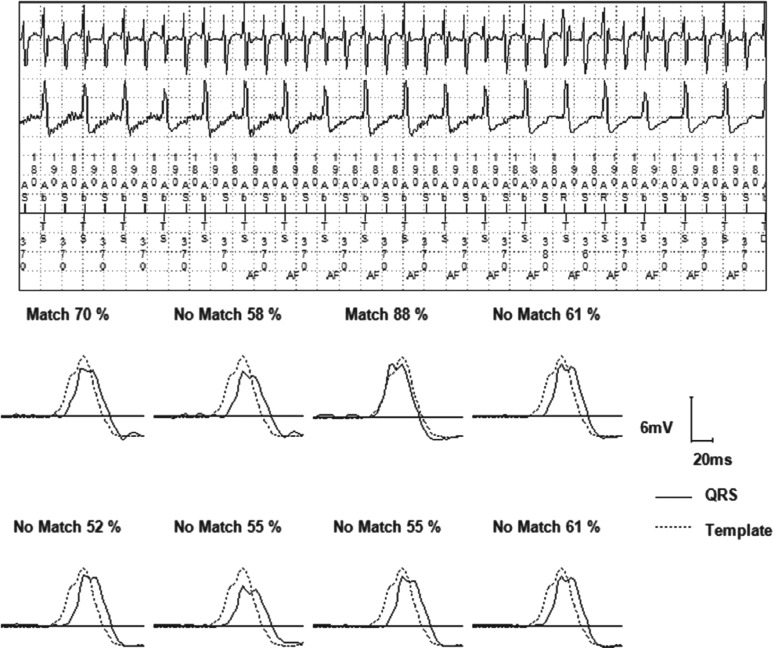
An example of inappropriate shock due to AF with a VT zone programmed at 370 ms due to previous monomorphic VT at this rate. In the top panel, PR Logic™ (Medtronic, Minneapolis, MN, USA) was used and AV dissociation was deemed to be present, likely due to the changing relationship between the atrial- and ventricular-sensed activities. The bottom panel shows that the morphology discriminator (Wavelet) detects that the QRS morphology is on average less than a 70% match for eight QRS complexes as compared with the template that was created. The rhythm is then deemed to be a double tachycardia with AF and VT as noted and antitachycardia pacing is subsequently delivered. Options to avoid repeat inappropriate therapy include decreasing the Match threshold for Wavelet. The VT zone could also be changed and the tachycardia onset discriminator could be turned off.

Various factors can lead to inappropriate shocks. The cornerstone of any ICD’s recognition of VT or VF is the patient’s ventricular rate. Thus, supraventricular tachycardias (SVTs) including sinus tachycardia, AF, and atrial flutter may provoke the occurrence of inappropriate shocks **(Figure 1)**. In other cases, device malfunction can result in unpredictable behavior and the erroneous detection of either VT or VF. In SCD-HeFT and MADIT-II, the most common causes of inappropriate shocks were, in descending order, AF; a combination of other SVTs including sinus tachycardia, atrial tachycardias, and paroxysmal supraventricular tachycardias; and, finally, oversensing caused by lead fracture, T-wave oversensing, and electromagnetic interference. The ALTITUDE-NOISE study demonstrated that, out of 1,570 inappropriate shocks, 8.5% were due to noise, artifact, and oversensing.^13^ Specifically, the episodes were determined to be due to external noise in 57%, lead-connector issues in 28%, muscle noise in 8%, atrial oversensing in 5%, and T-wave oversensing in 2% of participants, respectively.^13^ In a more recent study, independent predictors of inappropriate shocks included not only AF but also an age of younger than 70 years.^6^

## Preventing inappropriate shocks

The prevention of inappropriate shocks has been approached in multiple ways, including by way of various attempts to optimize device selection, ICD programming, remote monitoring, pharmacologic therapy, and catheter ablation.

### Device selection

It has been considered that the use of dual-chamber rather than single-chamber ICDs may help to reduce inappropriate shocks by allowing for better discrimination between AF/SVTs and VT/VF. Notably, some of the SVT discrimination techniques described below require the presence of an atrial lead. Earlier studies offered conflicting results regarding the benefit^14^ or lack thereof^15^ of an atrial lead for this purpose. However, multiple pooled analyses contradict the advantages of dual-chamber devices for this purpose.^16,17^ In one large review, among patients who received an ICD for primary prevention without indications for pacing, dual-chamber devices were not associated with a lower risk of inappropriate shock or differences in hospitalization or death as compared with single-chamber devices.^18^

### Rate and duration settings

There are several device settings that can help to prevent inappropriate therapy. The first step in triggering ICD therapy is the patient’s heart rate exceeding a programmed rate for a programmed duration. Setting a therapy zone only to shock for very fast rates may help to avoid shocks for SVT, and a therapy zone set for longer periods before detection of a ventricular arrhythmia may help bypass the administration of shocks for nonsustained VT. Prospective randomized trials have shown that programming the ICD to deliver therapy only for very fast rates and for longer durations may be beneficial. In MADIT-RIT, there were two arms that were compared to conventional settings **(Table 1)**. In one arm, a detection rate above 200 beats/min was used in an attempt to avoid inappropriate shocks. In the other arm, delays in therapy were programmed with the same intent. Both of these techniques appeared to provide benefit versus conventional settings. The benefit seen was not only a substantially lower rate of inappropriate shocks (HR: 0.21 as compared with conventional settings) but also a significantly lower rate of all-cause mortality (HR: 0.45).^10^ Similarly, in ADVANCE III, patients were randomized to standard-detection (18/24 intervals) or long-detection (30/40 intervals) algorithms **(Table 1)**. Those with a longer detection time had a lower rate of inappropriate shocks without an increased risk of syncope.^11^

### Supraventricular tachycardia discriminators to minimize inappropriate shocks

Following the successful detection of VT based on the rate and duration criteria being met, the next step is the correct institution of programmed algorithms to discriminate VT from other fast rhythms. Therapy may be withheld if certain parameters are met. For instance, if the rate has exceeded the rate limit but the rhythm is irregular or the electrogram looks very similar to the template of sinus rhythm, therapy might be withheld. These discriminators can thus be useful tools in attempting to prevent the dispensation of inappropriate therapy.^19^ For example, in MADIT-II, not having an active AF discriminator was associated with a higher risk of inappropriate shocks.^1^

The specifics of these discrimination algorithms generally vary with device type. Certain discriminators are programmable in all modern devices, including single-chamber devices, while more advanced discriminators may be specific to devices with an atrial lead or to certain products of specific manufacturers. As a general rule, setting discriminators to more aggressively minimize inappropriate therapy comes at a cost of increasing the risk of withholding appropriate therapy in the face of need.^19^ These discriminators must be well-understood by the clinician in order to incorporate them effectively and in the appropriate patients.

### Single-chamber discriminators

The first few discriminators that we will discuss were intended for single-chamber devices, but they may be used in dual-chamber devices as well. It is important to recognize that discriminators in Medtronic (Minneapolis, MN, USA) and Boston Scientific (Natick, MA, USA) devices are programmable only for rates in the VT zone. The VF zone will typically administer therapy without employing SVT discriminators. The following are several key SVT discriminators.

#### Tachycardia onset

This discriminator was designed to minimize shocks for sinus tachycardia. The basis is that most VT episodes begin with an abrupt increase in rate, while sinus rhythm generally accelerates gradually to tachycardia. Algorithms were designed to evaluate the acceleration of the ventricular rate and therefore discriminate between an abrupt increase and a gradual increase in rate.

While the specific programming of this discriminator varies among device manufacturers, the overall goal is the same. The algorithm will allow for the adjustment of the percentage difference between the rate present before and that seen after tachycardia onset. Programming in a lower-percentage cutoff will allow for greater sensitivity and less specificity in VT detection. Use of this discriminator may not be appropriate for patients with exercise-induced VT or those with sudden-onset SVTs. Tachycardia-onset discriminators are typically no longer used in isolation. When used, they are often now able to be overridden by other algorithms where appropriate.

#### Stability

**Figure 2:**
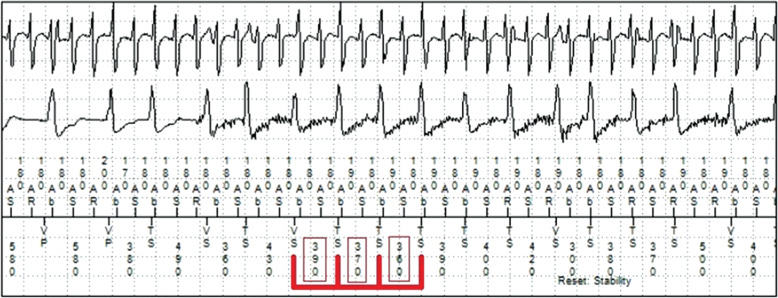
In this example, instability of the ventricular-sensed intervals is noted (red boxes) and therapy is appropriately withheld.

This discriminator was designed to minimize shocks for AF. The basis of this discriminator is that VT is typically a stable, regular rhythm, while AF conducts irregularly to the ventricle. The device attempts to withhold therapy if the coupling intervals are very irregular **(Figure 2)**.

When this discriminator is used, a programmable value that is designed to define instability must be set. The interval between VT beats is measured and, if enough intervals are greater than the programmed cutoff, indicating an irregular rhythm, then the VT counter is reset. Programming a longer interval will lead to greater sensitivity and less specificity for VT detection.

A limitation of this algorithm is that, during AF at faster rates, ventricular activity may appear more “regular” and may be mistaken for VT. Additionally, polymorphic VT that falls into the SVT discriminator zone could potentially be mistaken for AF. In some dual-chamber devices, this discriminator may be activated only if the atrial channel detects AF, which may improve on some of these limitations.

#### Waveform morphology

One factor discriminating SVT from VT is the electrogram morphology during tachycardia. VT will typically have a very different morphology than that seen during sinus rhythm, while that for SVT may be similar to that for sinus rhythm. This SVT discriminator was designed around this concept.

There are various methods by which devices can assess the QRS morphology and, over time, these methods have become more sophisticated. Contemporary morphology algorithms now store and analyze a sample of a waveform during the baseline rhythm, analyze a sample of the waveform during tachycardia, compare the baseline and tachycardia waveforms, and score this comparison as either similar enough to withhold therapy or different enough to continue with VT detection and treatment. While the more rudimentary forms of this discriminator may be used in single-chamber devices, the more advanced forms of morphology analysis are useful in dual-chamber devices and are similarly appropriate as one component of complex discriminators.

The Wavelet represents the contemporary form of this discriminator in Medtronic devices (Minneapolis, MN, USA). Generally, the device works by recording and storing a template of a normal QRS wave. During VT detection, the VT wave is compared with the stored template. The algorithm then quantifies a match-percentage score between the two. VT detection is withheld if three or more of the last eight QRS complexes match the template. The match-percentage score cutoff is programmable, with a nominal setting of 70%. If the percentage cutoff is decreased, there is an increase in the likelihood that the device will withhold detection appropriately, but a decrease in the likelihood that it will detect true VT (**Figure 1**, bottom panel).

Conversely, in Boston Scientific devices (Natick, MA, USA), this discriminator is called Vector Timing and Correlation. This algorithm also compares the timing of the shock electrogram with the timing of the local RV lead electrogram. In St. Jude Medical–branded (St. Paul, MN, USA; products now part of the portfolio of Abbott Laboratories, Chicago, IL, USA) devices, the algorithm compares the waveform in the context of number, sequence, polarity, amplitude, and area of waveform peaks to the stored template.

### Dual-chamber discriminators

The aforementioned discriminators were designed with single-chamber devices in mind, but they are available on most modern single- and dual-chamber ICDs. In comparison, more advanced discrimination algorithms are permitted when an atrial lead is utilized, by comparing the rates between the ventricular channel and the atrial channel. The specifics of these algorithms differ between device manufacturers, as follows.

**Figure 3:**
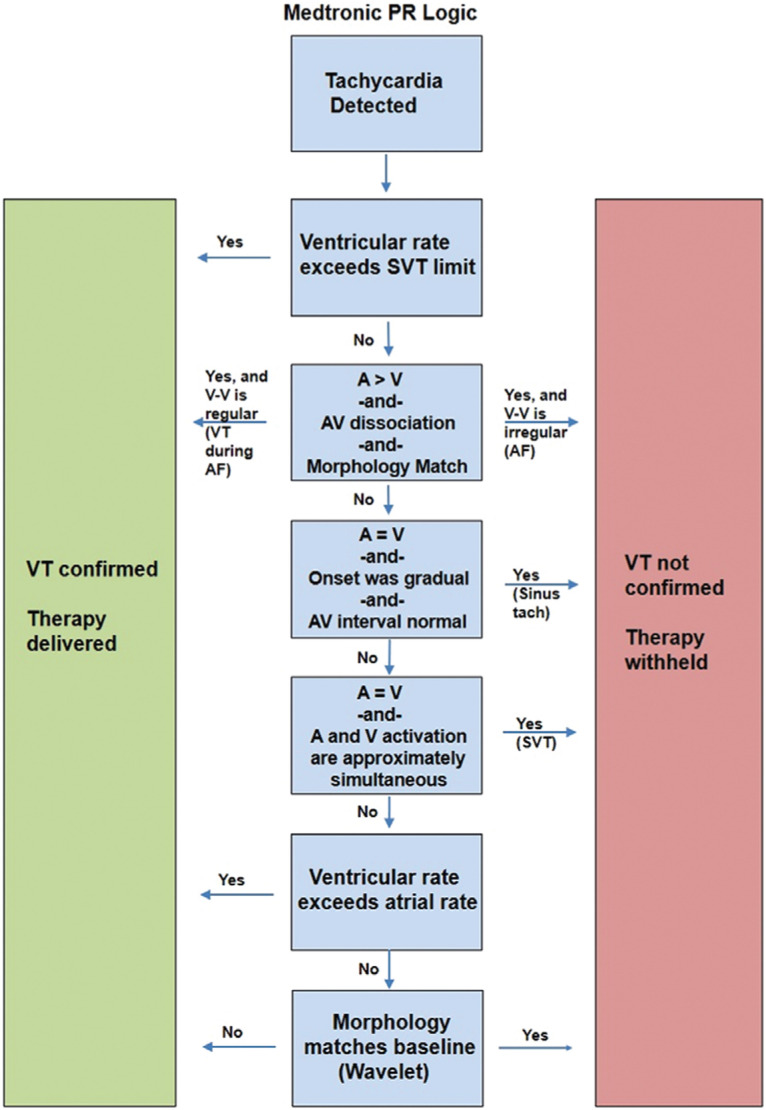
A simplified overview of the PR Logic™ algorithm (Medtronic, Minneapolis, MN, USA).

**Figure 4:**
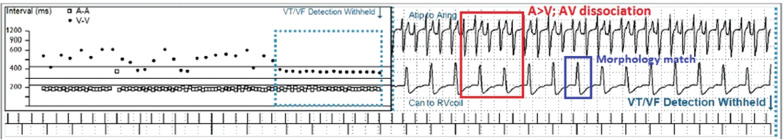
Following the PR Logic™ algorithm (Medtronic, Minneapolis, MN, USA), the atrial rate and ventricular rate are compared. Here, due to the fact that the atrial rate exceeds the ventricular rate and as there appears to be some degree of AV dissociation (red box) and also the morphology matches the template of sinus rhythm (blue box), therapy is appropriately withheld during AF.

Dual-chamber ICDs from Medtronic (Minneapolis, MN, USA) use a multistep algorithm called PR Logic™ **(Figure 3)**. PR Logic™ employs an analytical approach in the face of events sensed in the atrium as well as events sensed in the ventricle. The algorithm analyzes atrial and ventricular events and considers not only the rate and regularity but also the pattern of atrial events relative to ventricular events and atrioventricular (AV) association **(Figures 1 and 4)**. A primary goal of PR Logic™ is to determine if a double tachycardia (VT/VF in the presence of an SVT) is present by analyzing whether or not the ventricular rhythm is regular (for the VT zone only), looking for both evidence of AV dissociation (P–R interval fluctuation) and for evidence of AF (multiple atrial electrograms in the R–R interval). The Wavelet discriminator is then also incorporated into PR Logic™. PR Logic™ and Wavelet can be utilized even for tachycardias that demonstrate rates falling within the VF zone.

**Figure 5:**
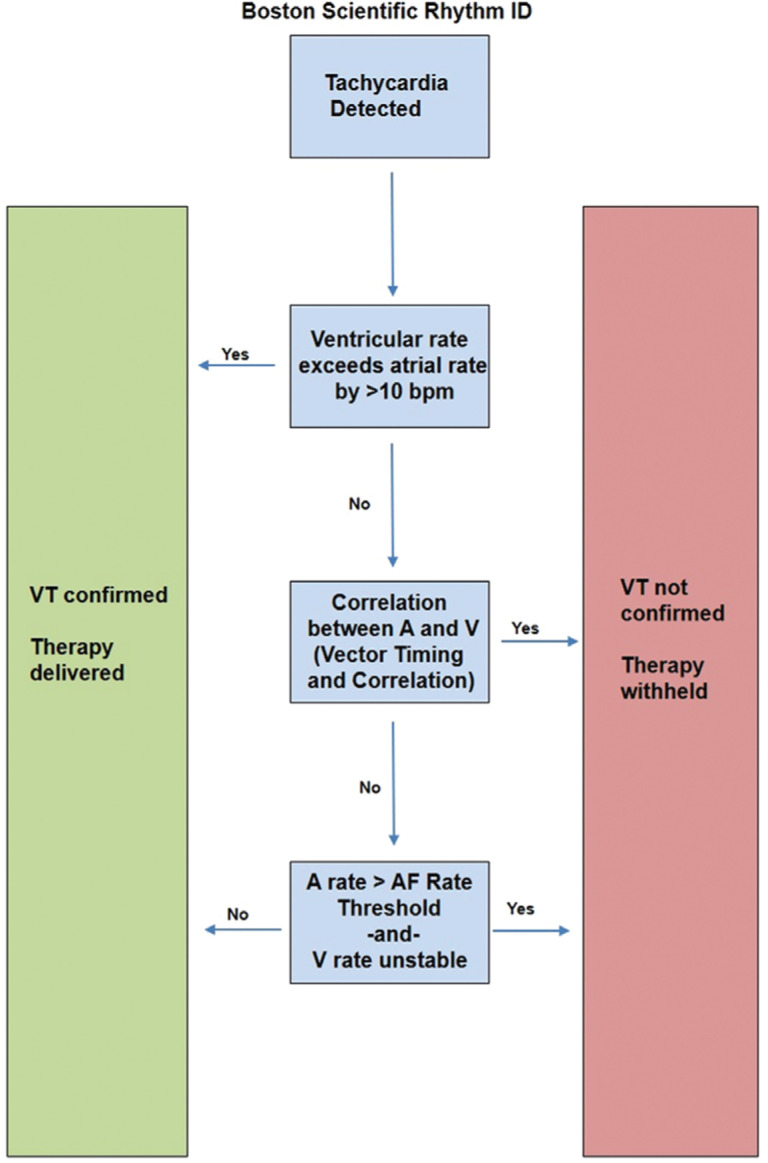
A simplified overview of the Rhythm ID algorithm (Boston Scientific, Natick, MA, USA).

**Figure 6:**
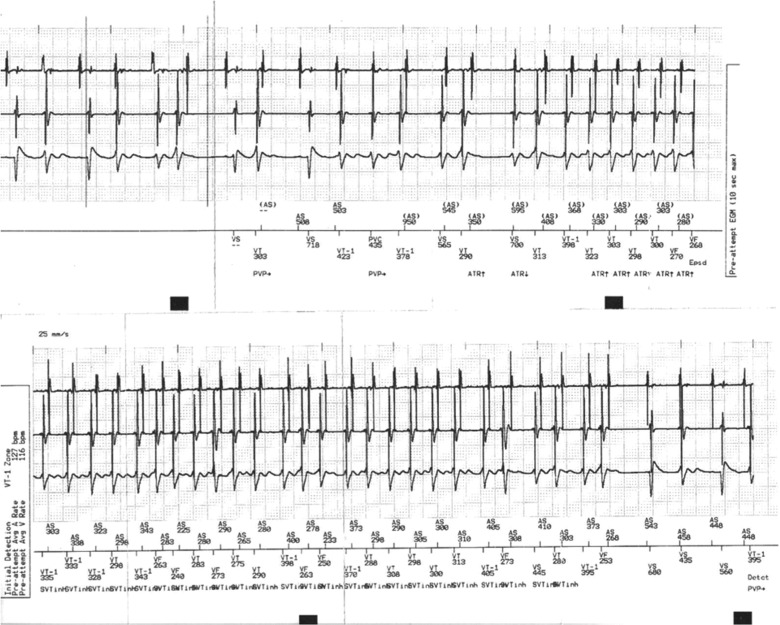
Example of Rhythm ID (Boston Scientific, Natick, MA, USA). In a typical case, first, there is a determination made regarding whether or not the ventricular rate is greater than the atrial rate; in this example, it is not. The next step is to compare the timing of the ventricular electrogram with the shock electrocardiogram and the morphology of the shock electrogram in tachycardia as compared with a baseline template to determine whether or not there is a sufficient match (the morphology match can be programmed between 70% and 96%). Finally, the algorithm determines if the atrial rate is greater than 170 bpm and if the ventricular rhythm is unstable (> 20-ms variability) in order to make the final determination of categorizing a rhythm as SVT or VT.

Boston Scientific devices (Natick, MA, USA) use a multistep discriminator called Rhythm ID with RhythmMatch™ **(Figure 5)**. Rhythm ID considers the ventricular rate in comparison with the atrial rate, analyzes the morphology using Vector Timing and Correlation, and uses these factors to discriminate VT from other arrhythmias **(Figure 6)**. The RhythmMatch™ component is a programmable correlation factor, intended to allow for the customization of a device’s discrimination sensitivity during reprogramming.

**Figure 7:**
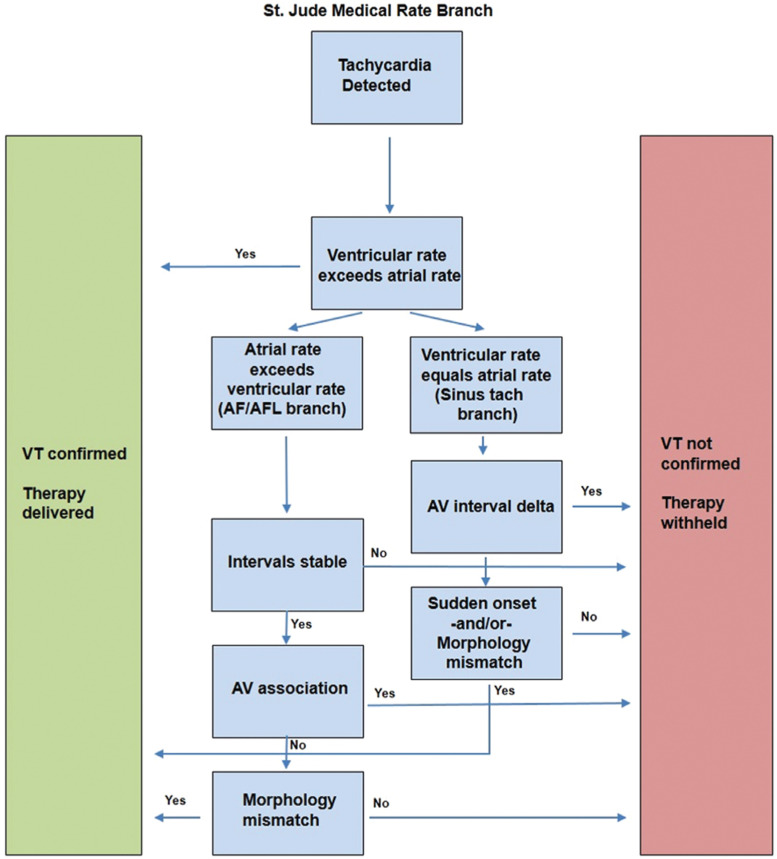
A simplified overview of the Rate Branch algorithm (Abbott Laboratories, Chicago, IL, USA).

Dual-chamber ICDs belonging to Abbott Laboratories (Chicago, IL, USA), including specifically St. Jude Medical–branded ones (St. Paul, MN, USA), use an algorithm known as Rate Branch **(Figure 7)**. Both atrial and ventricular events are considered. During device functioning, the median atrial rate and median ventricular rate are compared and part of a branched algorithm is adhered to, depending on which rate is greater. For example, if the ventricular rate is higher, then VT is confirmed. If the atrial and ventricular rates are the same or if the atrial rate is faster, then additional discrimination is needed to differentiate VT from sinus tachycardia, AF, or SVT. Further discriminators such as morphology and sudden onset are then used based on the branching algorithm. In the case of an atrial rate exceeding a ventricular rate, discriminators such as interval stability and morphology are used in an attempt to discriminate VT from AF and SVT. If the atrial rate matches the ventricular rate, then different discriminators—such as morphology, sudden onset, or the change in AV intervals—are used to differentiate sinus tachycardia from VT.

**Figure 8:**
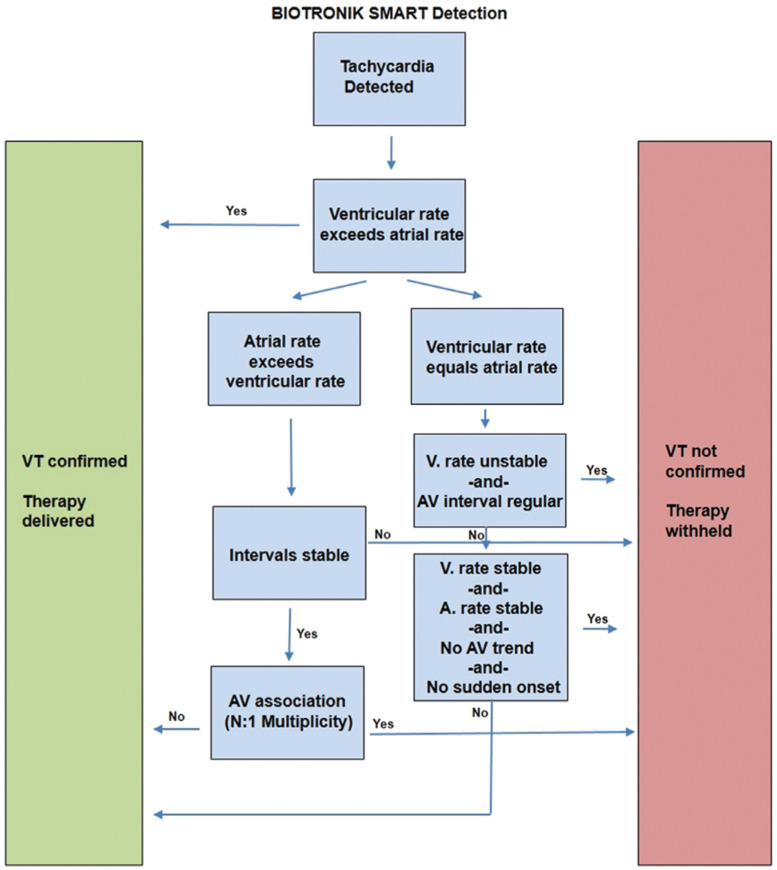
A simplified overview of the SMART algorithm (Biotronik, Berlin, Germany).

Biotronik (Berlin, Germany) devices use an algorithm known as SMART Detection™ **(Figure 8)**. This involves adherence to a branching set of algorithms, beginning with considering the relative rates of atrial- and ventricular-sensed events. Similar to Rate Branch, the relative atrial–ventricular rates are then sorted by other discriminators such as onset, stability, and AV relationship to help discriminate VT from various SVTs.

### T-wave discrimination and right ventricular lead noise algorithms

Medtronic devices (Minneapolis, MN, USA) additionally have algorithms designed to minimize the risk of inappropriate shocks due to T-wave oversensing. Some devices also have algorithms to minimize the oversensing of right ventricular lead noise, which may be helpful in many patients. These algorithms may be useful even in patients with complete AV block or other situations in which the SVT discriminators described above should be programmed off. In PainFree SST, the use of SmartShock avoided inappropriate therapy for T-wave oversensing in 98% of episodes.^12^

### Pharmacologic rate-control therapy

In addition to optimizing device programming, there exist other, therapeutic approaches that may help to prevent inappropriate shocks. Medications are a potential adjunctive therapy to decrease the risk of inappropriate shocks. β-blockers, for instance, may help in multiple ways. A slower ventricular rate in AF may have a lower risk of reaching the ICD therapy zone. β-blockers may also help by decreasing the sinus rate to similarly avoid reaching the therapy zone, and could potentially prevent other atrial arrhythmias that might otherwise lead to inappropriate therapy.

Different β-blockers may have different effects on inappropriate shock rate. In a retrospective analysis of data from MADIT-CRT, patients receiving carvedilol were observed to have a significantly reduced risk of inappropriate shocks in comparison with those treated using metoprolol.^20^

### Pharmacologic rhythm control and catheter ablation

As AF is an important cause of inappropriate shocks, limiting the burden of AF with antiarrhythmic medications or catheter ablation is an important consideration for patients with ICDs and AF. Patients with ICDs are often on antiarrhythmic therapy for arrhythmia management. Few studies have demonstrated an effect of antiarrhythmic medication on reducing inappropriate shocks.^21^ Catheter ablation should not be overlooked as an adjunctive strategy to decrease the risk of inappropriate shocks. Catheter ablation of SVTs and atrial flutter is often successful at eradicating these arrhythmias and should be considered for deployment in patients with ICDs. The ablation of AF should also be considered. In one cohort of 73 patients with ICDs undergoing AF ablation, a significantly lower rate of both appropriate and inappropriate shocks was seen after ablation as compared with prior to ablation.^22^

### Remote monitoring

While medications may help to reduce the risk of inappropriate shocks due to other arrhythmias, they will do little to prevent shocks due to noise and abnormal device function. One strategy here therefore is to use remote monitoring in an attempt to recognize abnormal lead function early and thus intervene quickly so as to prevent future shocks. In one recent study of ICDs with careful remote monitoring spanning 4,457 patient-years of follow-up, 95% of lead failures were diagnosed before any complications arose. Inappropriate shocks occurred with an annual rate of only 0.04% in this cohort.^23^

Additionally, remote monitoring may allow for an earlier diagnosis of SVTs or other arrhythmias, enabling more prompt treatment to be delivered and possibly avoiding some inappropriate shocks. One trial compared patients with remote monitoring with a control group participating in routine clinic visits every six months. First and subsequent inappropriate shocks and their causes were compared between the groups. Over a follow-up of 27 months, the rate of inappropriate shocks was 5% in the remote monitoring group as compared with 10.4% in the control group. Notably, the numbers of inappropriate shocks delivered per patient triggered by SVT and by lead dysfunction were 74% and 98% lower, respectively, in the remote monitoring group.^24^

Remote monitoring has even been shown to be associated with improved mortality and lower economic costs. In 2015, Varma et al. demonstrated an association between improved survival and higher rates of remote monitoring utilization among 269,471 patients with implanted cardiac devices.^25^ The next year, Piccini et al. showed that, among 92,566 patients with devices, those who utilized remote monitoring had a lower risk of hospitalization, a shorter hospitalization length, and a 30% reduction in hospitalization costs.^26^

As the importance of remote monitoring has increasingly become apparent, device companies have begun improving device capabilities with consideration of this feature. Biotronik (Berlin, Germany) devices were the first to allow for cellular connectivity with an integrated radiofrequency antenna that is capable of transmitting data quickly to providers via a wireless device carried by the patient. Newer devices by other companies are also beginning to incorporate enhanced wireless connectivity.

## Conclusion

Inappropriate ICD shocks remain a significant problem, demonstrating an increased association with morbidity, reduced patient quality of life, and greater health care utilization. However, with a better understanding of the causes of inappropriate shocks and the use of a thoughtful, multifaceted approach to incorporating device programming and adjunctive strategies, inappropriate shocks can be minimized.

It is reasonable at this time to rely on the ADVANCE III or either of the MADIT-RIT settings when programming devices, as both are evidence-based strategies aimed at minimizing inappropriate shocks as well as providing a benefit with regards to morbidity and mortality. We recommend remote monitoring for patients with devices whenever feasible. Finally, the treatment of any comorbid arrhythmias with pharmacologic or ablation strategies when appropriate is recommended to further assist in limiting inappropriate shocks.

## Figures and Tables

**Table 1:** Rate and Detection Cutoff Values Used in MADIT-RIT^10^ and ADVANCE III^11^

**MADIT-RIT (high-rate arm) settings**			
	**Rate Cutoff**	**Duration Cutoff**	**Therapy**
VT zone	170 bpm		Monitoring only
VF zone	200 bpm	2.5 seconds	ATP + shock
**MADIT-RIT (duration-delay arm) settings**			
	**Rate Cutoff**	**Delay**	**Therapy**
VT-1 zone	170 bpm	60 seconds	ATP + shock
VT-2 zone	200 bpm	12 seconds	ATP + shock
VF zone	250 bpm	2.5 seconds	ATP + shock
**ADVANCE III settings**			
	**Rate Cutoff**	**Detection Cutoff**	**Therapy**
VT zone	150 bpm		Monitoring only
VF zone	188 bpm	30/40 detection intervals	ATP while charging; shock

ATP: antitachycardia pacing; VF: ventricular fibrillation; VT: ventricular tachycardia.
